# Delta Power in SLC6A1-Related Neurodevelopmental Disorder: Operationalizing Quantitative EEG Metrics for Biomarker Development

**DOI:** 10.3390/neurolint18030058

**Published:** 2026-03-18

**Authors:** Hamza Dahshi, Marie Varnet, Kimberly Goodspeed, Jacob Tiller, Dallas Armstrong, Deepa Sirsi

**Affiliations:** 1 Medical Scientist Training Program, School of Medicine, Case Western Reserve University, 9501 Euclid Ave, Cleveland, OH 44106, USA; 2 O’Donnell Brain Institute, University of Texas Southwestern Medical Center, 5323 Harry Hines Blvd, Dallas, TX 75390, USA; 3 Department of Neurology, University of Texas Southwestern Medical Center, 5323 Harry Hines Blvd, Dallas, TX 75390, USA; marie.varnet@cuanschutz.edu (M.V.); dallas.armstrong@utsouthwestern.edu (D.A.); deepa.sirsi@utsouthwestern.edu (D.S.); 4 Ultragenyx Pharmaceutical Inc., 60 Leveroni Court, Novato, CA 94949, USA; 5 Division of Biology and Medicine, Department of Neuroscience, Brown University, 1 Prospect St, Providence, RI 02912, USA; jacob_tiller@brown.edu; 6 SLC6A1 Connect, 1939 Temperence Hill Drive, Frisco, TX 75034, USA

**Keywords:** EEG, Epilepsy, delta power, *SLC6A1*

## Abstract

Introduction: SLC6A1-related neurodevelopmental disorder (SLC6A1-NDD) is an epileptic encephalopathy linked to mutations in the *SLC6A1* gene and is characterized by early-onset seizures and developmental delays. Despite the growing recognition of *SLC6A1* as a major cause of early-onset epilepsy, the electrophysiological changes associated with the disorder remain inadequately characterized. This study aims to identify electrophysiological biomarkers of SLC6A1-NDD by characterizing EEG delta power using automated tools, EEGLAB (v2023.1) and Persyst 13, exploring age- and state-related effects. Methods: We analyzed EEG recordings from 20 patients with SLC6A1-NDD and 20 neurotypical age- and sex-matched controls using EEGLAB and Persyst, quantifying delta power and related metrics. The Wilcoxon signed-rank method tested for differences between patients and controls, area under the curve (AUC) values evaluated patient classifier models, and Pearson’s correlation assessed concordance between EEGLAB and Persyst. Results: Patients with SLC6A1-NDD exhibited significantly elevated delta power (19.4 ± 4.1) compared to controls (14.2 ± 3.0; *p* < 0.001). The mean delta power showed an age-dependent increasing trend in patients (b = 0.5), contrasting with a decline in controls (b = −1.0; *p* < 0.001). In Persyst, the frequency of delta activity above an optimized threshold best differentiated patients from controls in wake epochs (AUC = 0.93). Concordance between EEGLAB and Persyst was one-to-one but with moderate variability (R^2^ = 0.644; *p* < 0.001). Conclusions: Elevated delta power is a notable feature of SLC6A1-NDD. Cross-platform comparison demonstrates the feasibility of quantitative EEG analysis, while imperfect concordance highlights the need for pipeline standardization. Future work should validate these findings in larger cohorts and, as suitable reference data emerge, benchmark delta power metrics against age-matched children with other developmental and epileptic encephalopathies.

## 1. Introduction

Developmental epileptic encephalopathies (DEEs) are severe neurodevelopmental disorders marked by early-onset seizures, developmental delays, and abnormal EEG patterns. SLC6A1-related neurodevelopmental disorder (SLC6A1-NDD) is a rare DEE linked to mutations in the *SLC6A1* gene, which encodes GABA transporter-1 (GAT-1) [1]. These mutations disrupt GABA reuptake, heighten neuronal excitability, and lead to seizures, intellectual disability, and behavioral challenges [2,3]. The electrophysiological changes in SLC6A1-NDD, however, remain poorly understood. The identification of biomarkers of SLC6A1-NDD could enable earlier diagnosis and serve as a target for novel therapies.

EEG is a routine tool in the diagnosis and understanding of genetic epilepsies. Quantitative EEG (qEEG) mathematically compresses raw traces into spectral power values for each frequency band, allowing large datasets to be reviewed at a glance. qEEG has been used to detect seizures and to monitor cerebral ischemia in both children and adults. It has also been used in recent biomarker work in Angelman syndrome for assessing therapeutics [4].

Delta power, representing the power spectral density within the 1–3 Hz frequency range, is relevant in other neurodevelopmental disorders and reflects disrupted cortical maturation [5]. Although delta power is elevated in many settings—normal deep sleep, early infancy, diffuse encephalopathy, or focal white matter injury—persistent delta excess during wakefulness has proven useful for flagging developmental channelopathies. Elevated delta power serves as a validated biomarker in genetic epilepsies such as Angelman syndrome [5,6]. Observations of intermittent rhythmic delta activity (IRDA) in routine EEGs of children with SLC6A1-NDD as part of the natural history study prompted systematic quantification.

Qualitative data on IRDA in SLC6A1-NDD is limited. The largest series to date (52 EEGs) found generalized epileptiform discharges in 69% of SLC6A1-NDD recordings; 23% showed 2–4 Hz spike-and-wave patterns often exacerbated by hyperventilation and accompanied by IRDA, typically maximal over the occipital region [1,3]. To date, however, no study has quantified IRDA or delta power objectively—emphasizing the need for the automated approach presented here.

Modern analysis platforms such as EEGLAB (an open-source MATLAB toolbox) and Persyst (FDA-cleared software for automated seizure detection) may permit the precise quantification of IRDA, enabling standardized and reproducible datasets. Automated EEG analyses for SLC6A1-NDD remain understudied, and rigorous cross-platform validation is needed. Furthermore, age, genetic background, and clinical state (wakefulness) can significantly influence EEG metrics [7,8,9]. Typically, delta power declines with age and during wakefulness, and its alteration can indicate developmental deviation. Investigating age- and state-related patterns in SLC6A1-NDD could improve biomarker specificity.

In this proof-of-concept study, we systematically characterize delta power in SLC6A1-NDD, compare it with age- and sex-matched neurotypical controls, and test how age and wake–sleep state modulate this signal. Using two complementary toolsets—EEGLAB and the FDA-cleared Persyst platform—we also quantify cross-platform concordance to establish a foundation for standardized, quantitative, automated EEG biomarkers in SLC6A1-NDD.

## 2. Methodology

### 2.1. Patients

This study included 20 patients with genetically confirmed SLC6A1-NDD and 20 neurotypical age- and sex-matched controls. The objective of this Phase 1 biomarker study was to assess whether quantitative EEG measures differentiate individuals with SLC6A1-NDD from matched typically developing controls [10].

Participants were enrolled in an ongoing natural history study and underwent annual visits; only first-visit data was analyzed. As an embedded proof-of-concept analysis within a prospective natural history study (annual 4 h EEGs for up to five years), the sample size was defined by available baseline EEG data at the time of analysis. This exploratory approach was intended to assess feasibility and signal detection rather than to provide definitive estimates of effect size. Parent interviews and clinical record reviews provided detailed information on seizure history, medication use, and developmental concerns [11,12]. Pathogenic, likely pathogenic, or phenotype-consistent variants of unknown significance (VUS) in *SLC6A1* were verified by clinical genetic reports.

Controls were drawn from children referred for spell evaluation whose EEGs were (1) read as normal and (2) whose medical records lacked any neurological or developmental diagnosis. Using healthy controls allowed us to establish the disease-vs.-health signal-to-noise ratio for delta power—an essential first step prior to larger Phase 2 studies that will compare SLC6A1-NDD with other DEEs and medication profiles [10]. This phased approach mirrors biomarker development standards in oncology and neuroimaging.

All recordings were 4 h outpatient video EEGs (Natus Xltek NeuroWorks (Natus Neurology Incorporated, Oakville, ON, Canada); 256 Hz sampling; 10–20 montage plus single-lead ECG). A board-certified pediatric epileptologist evaluated all EEGs and visually scored wake versus sleep epochs. Each control EEG was confirmed normal by the same epileptologist and matched 1:1 to a patient by age (±6 months) and sex. Exclusion criteria for both groups included metabolic disorders, acute encephalopathy, or technical EEG artifacts. Sleep stage comparisons were limited by the incomplete matching of sleep states between patients and controls. Because available recordings did not consistently include corresponding sleep stages across matched pairs, direct statistical comparisons of sleep state delta power between groups were not performed.

### 2.2. EEGLAB Software

EEGLAB is an open-source MATLAB toolbox and graphical user interface for processing multichannel EEG data, supporting both single-trial and averaged analyses across any number of channels [13]. Power spectral density estimates were derived, in decibel units (dB), via the EEGLAB toolbox using the entire EEGs. To calculate delta power for each channel, we averaged the dB values from 1 to 3 Hz. The mean delta power was the average across all channels. Default settings in EEGLAB did not separate awake and sleep states. Portions of the EEG containing artifacts were excluded using EEGLAB’s automated artifact rejection plugin.

### 2.3. Persyst Software

Persyst 13 (Persyst Development Corporation, Persyst Development Corporation, Solana Beach, CA, USA) is an FDA-cleared software that offers a range of quantitative EEG trend analyses, including power spectral displays, spike detection, and seizure probability mapping. Delta band power (1–3 Hz) was exported from Persyst in contiguous 8 s epochs for all cortical channels (linear units, µV^2^/Hz), in contrast to EEGLAB which defaults to dB for units. An 8 s epoch captures 8–24 oscillatory cycles within the 1–3 Hz delta band, providing stable spectral power estimation while maintaining clinically relevant temporal resolution. From these values, we derived four delta power-based metrics:

1. Minimum differentiating threshold (µV^2^): We first sought to determine whether there is a single delta power value that best separates patients from controls. To test this, candidate thresholds were evaluated in 1 µV^2^ increments. For each candidate threshold, a participant was classified as positive if their EEG contained at least one 8 s epoch in which any channel exceeded that value. Sensitivity and specificity were recalculated at each step, and the threshold that maximized Youden’s index (sensitivity + specificity − 1) was retained. Therefore, this metric metric evaluates whether the presence of any suprathreshold delta activity differentiates groups.
2. Total threshold-crossing frequency: We next extended this approach by allowing both the delta power threshold and the required number of suprathreshold events to vary. For each candidate delta power threshold, we counted how many 8 s epochs contained at least one channel exceeding that threshold. We then evaluated combinations of threshold value + minimum number of crossings to determine which pair best separated patients from controls (e.g., “>1000 µV^2^ crossed > 5 times”) via the area under the curve (AUC) score. Unlike Metric #1, which requires only a single suprathreshold event, this metric captures the burden of elevated delta activity across the recording.
3. Hourly threshold-crossing rate: Because EEG durations varied slightly across participants, the total number of threshold-crossing events was divided by recording duration (hours) to obtain a time-normalized crossing rate.
4. Percent channel-time above threshold: To quantify the overall proportion of EEGs occupied by elevated delta activity, we calculated the percentage of channel-time exceeding a given threshold. This was computed as (number of suprathreshold epochs × 8 s) ÷ total recording time, normalized by the number of channels. This metric reflects the fraction of possible channel-seconds during which delta power exceeded the selected threshold.

To have a comparable metric to EEGLAB, we expressed the mean delta power on Persyst in dB units. All metrics were calculated for the entire EEG and for wake epochs only, with Persyst automatic artifact reduction enabled as per vendor recommendations. These power-based surrogates serve as fully automated proxies for visually scored IRDA bursts and will require event-level validation in future work.

### 2.4. Electrode Groupings and EEG Processing Parameters

Specific electrodes were grouped into regions based on IRDA that was recognized by the epileptologists on qualitative EEGs performed for SLC6A1-NDD patients:

- Frontal (FIRDA): Fp1–F7, Fp2–F8.
- Occipital (OIRDA): P7–O1, P8–O2.
- Temporal (TIRDA): F7–T7, F8–T8.
- Midline: Fz–Cz.

These groupings align with prior studies investigating IRDA patterns in SLC6A1-NDD populations [14,15].

To enhance transparency in cross-platform comparison, key processing parameters are listed below for EEGLAB:

- Version: v2023.1; MATLAB R2024a.
- Sampling rate: 256 Hz (native acquisition rate).
- Delta band analyzed: 1–3 Hz.
- Power spectral density estimation: EEGLAB default spectopo function (Welch’s method).
- Artifact handling: EEGLAB built-in automated artifact rejection procedures (no external plugins applied).
- Referencing: Longitudinal bipolar montage; no additional re-referencing was applied within EEGLAB.
- Channels analyzed: All cortical channels (10–20 montage).

In comparison, the parameters for Persyst were as follows:

- Version: Persyst 13.
- Delta band definition: 1–3 Hz.
- Epoch length: 8 s (default trend configuration).
- Artifact handling: Persyst automatic artifact reduction enabled (vendor default).
- Referencing: Longitudinal bipolar montage.
- Channels analyzed: FIRDA, OIRDA, TIRDA, and midline groupings.

Because Persyst is proprietary software, internal filtering kernels and artifact reduction algorithms are not fully disclosed. Differences in filtering implementation, implicit referencing strategies, and artifact detection criteria may therefore contribute to observed cross-platform variance.

### 2.5. Statistical Analysis

Wilcoxon signed-rank tests compared delta power between patients and matched controls. Linear regression predicted delta power using age, and an ANOVA compared differences in trends between patients and controls. XGBoost Version 3.0, a gradient boosting decision tree classifier capable of handling non-linear multivariate relationships, classified disease status using delta power and age. Pearson’s correlation assessed concordance between EEGLAB and Persyst. Receiver operating characteristic (ROC) curves assessed the performance of quantified metrics. All analyses were performed using R Statistical Software (v4.4.0; R Core Team 2024) and EEGLAB toolbox (v.2023.1) via MATLAB (v24.1; R2024a) [13,16,17].

### 2.6. Ethical Approval and Consent

This study was approved by the UTSW Institutional Review Board (IRB; protocol code STU-2019-1561 and date of approval 2 December 2019). Written informed consent was obtained from all participants or their legal guardians.

## 3. Results

### 3.1. Patient Demographics

Patient demographics and clinical characteristics are summarized in Table 1. The SLC6A1-NDD cohort of 20 patients included an equal sex distribution and an average age of 5.3 years. Genetic testing was performed as part of clinical diagnostic evaluations with methods including single-gene and/or whole-exome sequencing, microarray and/or methylation analysis, and commercial sequencing panels. Most variants were de novo pathogenic missense mutations (*n* = 12/20, 60%). Absence seizures (*n* = 18/20, 90%) were the most prevalent epileptic presentation. Patients were on 0 to 4 (average of 1.3) anti-seizure medications (ASMs) during the EEG with Valproate being the most common ASM.

### 3.2. EEGLAB Analysis

**Figure 1:**
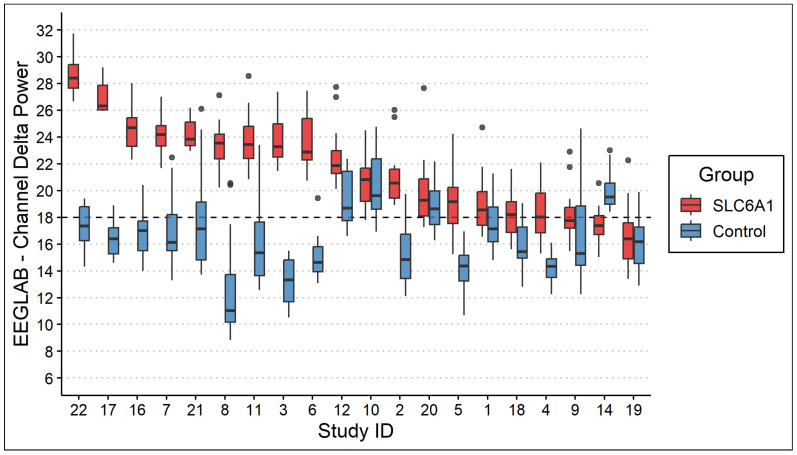
The boxplot distribution of EEGLAB delta power across EEG channels for SLC6A1-NDD patients and controls. Each channel’s delta power is a single value, and each box represents the distribution of those values for a single individual. Most SLC6A1-NDD patients (red) exhibited higher mean delta power compared to controls (blue; *p* < 0.001). The dashed line indicates the threshold of 18 μV^2^ identified through ROC analysis as having a sensitivity of 0.90 and specificity of 0.80 for differentiating patients from controls. Outliers are represented by gray dots.

Analysis showed 19 patients (95%) exhibiting higher mean delta power (19.4 ± 4.1) than controls (14.2 ± 3.0), with 15 (75%) showing statistical significance (*p* < 0.001; Figure 1). ROC analysis showed that a delta power > 18 has an AUC of 0.9125, a sensitivity (SN) of 0.90, and a specificity (SP) of 0.80. Three of the five patients with no significant delta power elevation were all less than 3 years old. This likely reflects normal developmental EEG physiology, as delta power is physiologically elevated in infancy and early childhood and typically declines with cortical maturation. Consequently, disease-related delta elevation may be less distinguishable from age-expected background activity in children under three years of age. Linear regression predicted an increase of 0.5 units in delta power per year of age in patients with SLC6A1-NDD, compared to a decline of 1 unit per year in controls (R^2^ = 0.60). The ANOVA determined a statistically significant difference between these two linear models (*p* < 0.001). Given the multivariate relationship, an XGBoost model classified patient status using both age and delta power; evaluation showed an AUC of 0.9875, SN of 94.5%, and SP of 86.3%.

### 3.3. Concordance Between Platforms

**Figure 2:**
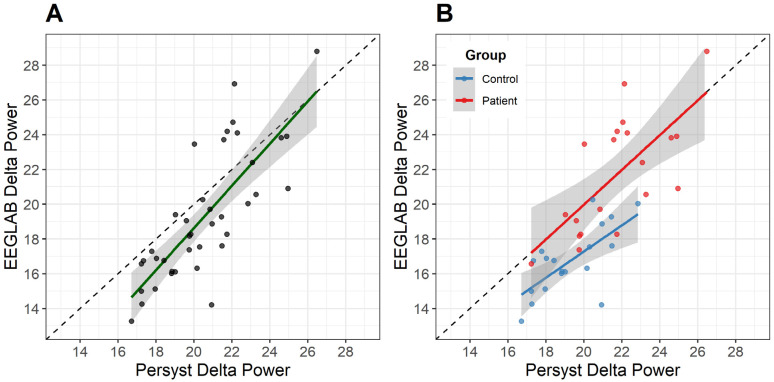
Concordance between EEGLAB and Persyst delta power across FIRDA, OIRDA, TIRDA, and midline channels. (**A**) Scatterplot showing statistically significant positive correlation (R^2^ = 0.644, *p* < 0.001) between EEGLAB and Persyst delta power across all participants. Dashed line represents line of identity, and solid green line represents best-fit regression line with confidence intervals shaded in gray. (**B**) Scatterplot stratified by group, with SLC6A1-NDD patients (red) exhibiting stronger one-to-one concordance between platforms compared to controls (blue) but with slightly higher variation. Solid red and blue lines represent best-fit regression lines for patients and controls, respectively, with 95% confidence intervals shaded in gray.

Pearson’s correlation showed a one-to-one correlation between EEGLAB and Persyst mean delta power. The best correlation was achieved when limiting Persyst channels to IRDA-related regions (R^2^ = 0.64, *p* < 0.001; Figure 2). Thus, all our further data analysis on Persyst-derived data was conducted on these channels only. Patient datapoints correlated more one to one, whereas controls showed higher delta power on Persyst compared to EEGLAB.

### 3.4. Persyst Analysis

We extracted delta power in the awake-only state and found that it was significantly elevated in patients (21.7 ± 2.33) compared to controls (19.1 ± 1.89) (*p* < 0.001). Although most patients (12/20) and controls (11/20) had their sleep states recorded on EEG, only five patients had corresponding age-matched controls with available sleep data. In addition, the recorded sleep stages differed both among patients and between patients and controls. Since different sleep stages are associated with varying physiological levels of delta power, with the highest delta power in N3 sleep, we were unable to perform a statistical comparison to assess differences in delta power between patients and controls.

**Figure 3:**
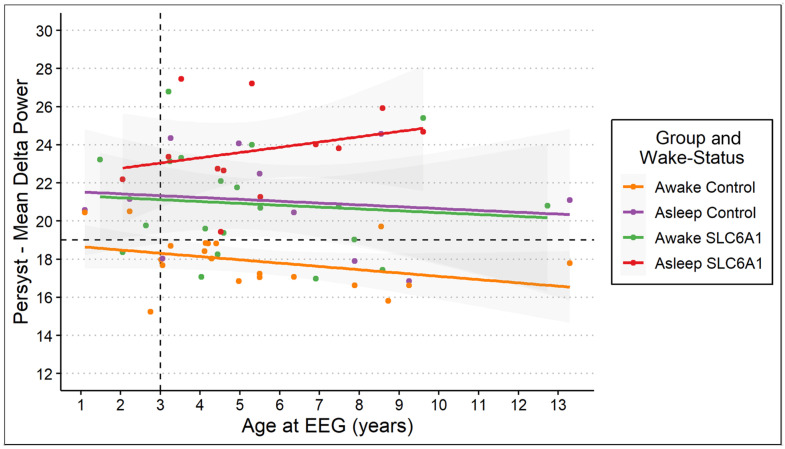
The linear regression of Persyst delta power as a function of age at EEG for awake and asleep states in SLC6A1-NDD patients and controls. Delta power increases significantly with age in the asleep state for patients (red) while remaining stable in asleep controls (purple). In the awake state, delta power decreases with age in controls (orange) and shows stability in patients (green), similarly to that of the asleep state in controls. The dashed horizontal line represents the delta power threshold of 19 μV^2^ identified in ROC analysis, and the dashed vertical line marks age 3 years, highlighting the age below which delta power differences are less pronounced. Shaded regions represent the 95% confidence intervals for each regression line. Of note, there are no wake and sleep measurements for each patient.

Multiple linear regression predicting delta power using age and wake state showed consistent trends with the EEGLAB linear model. Although matched patient–control pairs with sleep data were limited, linear regression allowed us to model age and state effects across the full sample, regardless of pairing. Notably, the sleep state trend in controls mimicked the awake state trend in patients; the ANOVA confirmed statistical significance (*p* < 0.001; Figure 3).

**Figure 4:**
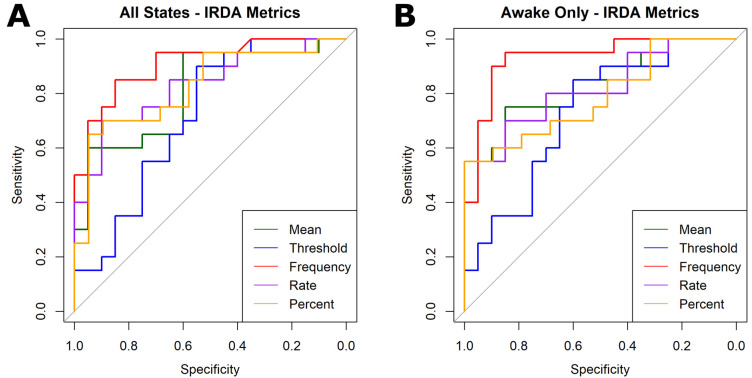
Receiver operating characteristic (ROC) curves for differentiating SLC6A1-NDD patients from controls using delta power-based metrics for (**A**) all sleep and wake states and (**B**) the awake-only state. Metrics include the (green) mean delta power, (blue) minimum differentiating threshold, (red) total frequency of threshold-crossing activity, (purple) hourly rate of threshold-crossing activity, and (orange) percent of EEG spent above threshold. Grid search techniques for maximizing the AUC determined optimal thresholds of 860 μV^2^ for all states and 86 μV^2^ for awake-only epochs. The frequency of threshold-crossing activity had the highest AUC values in all states (0.91) and awake-only epochs (0.93); however, this variable is not normalized to varying lengths of EEGs. Among the normalized variables, the hourly rate of threshold-crossing activity had the highest AUC in all states (0.83), while the mean delta power across the entire EEG best classified in the awake-only state (0.83).

Key ROC metrics are summarized in Table 2 and Figure 4. Across all sleep and wake epochs, the total threshold-crossing frequency performed the best with AUC values > 0.90; however, varying EEG lengths can bias this metric. Thus, we tested normalized metrics and found that the hourly crossing rate performed the best (AUC = 0.83) across entire EEGs, whereas the mean delta power outperformed (AUC = 0.83) in the awake-only state. Notably, percent time above threshold had relatively high specificity in both wake states, while the minimum differentiating threshold yielded the lowest AUC values.

## 4. Discussion

### 4.1. Key Findings

Our study demonstrates that patients with SLC6A1-NDD exhibit significantly elevated delta power compared to age- and gender-matched controls. This feature was observed across both EEGLAB and Persyst, with strong concordance between the two methods. Notably, we observed increased delta power in the SLC6A1-NDD cohort. It also aligns with prior findings that identified elevated delta power as a hallmark of SLC6A1-NDD and as a potential indicator of abnormal neural synchronization in genetic epilepsies [18,19,20]. Automated delta power quantification converts subjective background-slowing into a single objective value that EEGLAB or Persyst can compute efficiently. This reproducible metric could facilitate cross-center comparisons, flag EEGs for targeted *SLC6A1* testing, and—pending longitudinal validation—support future treatment response monitoring.

Furthermore, delta power showed an increasing trend with age in SLC6A1-NDD patients, contrasting with the decline observed in controls. We also observed that the age-dependent trend in the asleep state of controls parallels the trend in the awake state of SLC6A1-NDD patients. These age-dependent trends highlight potential neurodevelopmental differences in the SLC6A1-NDD population as previous research emphasized age-related changes in delta power as a reflection of neural maturation [5,7,8,19,21]. These disruptions in typical age-dependent trends may underlie the clinical features of SLC6A1-NDD such as developmental delay, regression and seizures [3,18]. It is crucial to further our understanding of delta power as a potential diagnostic and developmental biomarker.

Delta power-derived metrics using Persyst showed promise as quantitative indicators for distinguishing patients from controls. Most metrics achieved AUCs > 0.8, in the awake state. These findings bridge the gap between qualitative IRDA observations in traditional EEG analyses and fully automated power surrogates.

### 4.2. Clinical Implications

The identified delta-related metrics provide a foundation for objective biomarkers that could complement traditional EEG review. Biomarkers that are relatively readily available to track and measure are especially valuable when establishing pharmaceutical and gene therapy.

Interrater reliability for rhythmic patterns on EEG is only moderate [22]. Persyst’s high sensitivity in detecting delta power-derived metrics supports its use as a scalable and standardizable tool for clinical and research settings. Future studies may manually annotate IRDA bursts visually and validate its quantitative values with these automated surrogate markers.

### 4.3. Limitations and Opportunities

Our initial limitations revolved around the data itself. The single-center sample (*n* = 20 SLC6A1-NDD) limits external validity. Ongoing multicenter recruitment would refine age-normed delta power cut-offs and allow for medication-stratified analyses. Furthermore, our study did not control for ASM use, psychiatric symptoms, or epilepsy, all of which are known to influence EEG patterns [23,24,25]. Sleep state analysis was also limited by the lack of corresponding age-matched controls with available sleep data in similar stages of sleep. Physiological delta power varies with different sleep stages, with the highest delta power in N3 sleep. Without corresponding sleep data, we were not able to perform statistical comparisons between patients and controls. This limitation highlights the need for future studies to address this question more systematically.

We intentionally used neurotypical controls rather than other DEEs to establish a clear disease-vs.-health signal in this first-step biomarker study. Given that (1) normative delta power trajectories across childhood are well-described, whereas comparable reference data for most rare DEEs are lacking, and (2) SLC6A1-NDD already exhibits substantial clinical heterogeneity and poly-therapy confounding, broadening the comparison set at this early stage would have obscured the biological signal we sought to quantify. Importantly, our aim was not to declare delta power pathognomonic for SLC6A1-NDD but to demonstrate that an objective, automated EEG metric can capture the disease-relevant slowing that clinicians report qualitatively.

EEGLAB and Persyst produced a 1:1 relationship but only moderate concordance (R^2^ = 0.64). This likely reflects hidden differences in filtering, artifact rejection, and referencing. Because the aim here was feasibility rather than optimization, we accepted this variance; nonetheless, the findings highlight the need for transparent, standardized pipelines. Proprietary constraints currently limit the full validation of Persyst algorithms.

### 4.4. Future Directions

Longitudinal data from the SLC6A1-NDD natural history study will enable us to explore how delta power and delta power-derived metrics evolve and correlate with clinical outcomes such as seizure frequency, developmental regression, cognitive development, maladaptive behavior, and motor function.

Building on these findings, our XGBoost model demonstrated excellent performance using age and delta power on a sample size of 40. XGBoost is a gradient-boosted decision tree algorithm well-suited for modeling complex, non-linear interactions between multiple variables. We intentionally limited multivariate modeling to delta power alone; this decision reflects this study’s primary aim as a proof of concept. Testing various models with different metrics would have added complexity without increasing interpretive or diagnostic value at this stage. Additionally, limiting our model to two variables aligns with the well-used “rule of thumb” requiring a sample size of at least 10 events per candidate predictor [26]. Expanding this approach to include a larger sample size could allow for model validation on test sets, improving generalizability.

## 5. Conclusions

Elevated delta power is an electrophysiological feature of SLC6A1-NDD that can be quantified on clinically available platforms. Automated measurement offers a practical first step toward an objective EEG biomarker, even though cross-platform variance highlights the need for pipeline standardization. Future studies should validate these metrics in larger multicenter cohorts and, as suitable reference data emerge, benchmark them against age-matched children with other developmental epileptic encephalopathies while tracking longitudinal clinical outcomes and refining fully automated workflows.

## Figures and Tables

**Table 1:** Demographic and clinical characteristics of SLC6A1-NDD patients vs. controls.

Characteristic	SLC6A1-NDD (*n* = 20)	Controls (*n* = 20)
**Demographics**		
Age, years—Range	1.5–12.7	1.1–13.3
Age, years—Mean ± SD	5.3 ± 2.8	5.4 ± 2.9
Sex (Male:Female)	10:10	10:10
**Anti-Seizure Medications (ASMs)**		
Number of Prior ASMs (Mean ± SD, Range)	3.9 ± 2.6 (0–9)	N/A
Most Common Previously Prescribed ASM	Levetiracetam (11/20)	N/A
Concurrent ASMs at Enrollment (Mean ± SD, Range)	1.3 ± 1.2 (0–4)	N/A
Most Common Current ASM	Valproate (7/20)	N/A
**Genetic Findings**		
Inheritance	12 de novo, 2 inherited, 6 unknown	N/A
Mutation Type	16 missense, 3 nonsense, 1 non-coding	N/A
Variant Classification	12 pathogenic, 5 likely pathogenic, 3 VUS	N/A
**Seizure Types**		
Absence	18 (90%)	N/A
Atonic	13 (65%)	N/A
Myoclonic–Atonic	6 (30%)	N/A
Myoclonic	4 (20%)	N/A
Generalized Tonic–Clonic (GTC)	1 (5%)	N/A
≥2 Seizure Types	17 (85%)	N/A
Age at Seizure Onset (years, Mean ± SD)	1.64 ± 0.69	N/A
**EEG and Development**		
Age at First Abnormal EEG (years, Mean ± SD; Median)	3.0 ± 1.4; 2.8	N/A
Age at Initial Developmental/Behavioral Concerns (months, Mean ± SD, Range)	7.6 ± 6.7 (1–24)	N/A

**Abbreviations:** ASM, Anti-Seizure Medication; VUS, Variant of Unknown Significance; N/A, Not Applicable.

**Table 2:** Delta power-derived ROC metrics for differentiating SLC6A1-NDD patients from neurotypical controls.

Metric	EEG State	Optimal Threshold	AUC	Sensitivity	Specificity
Mean Delta Power	All States	>19 dB	0.8075	0.95	0.60
Mean Delta Power	All States	>21.5 dB	0.8075	0.60	0.95
Mean Delta Power	Awake	>19 dB	0.8325	0.75	0.85
Minimum Differentiating Threshold	All States	>4256 μV^2^	0.7175	0.90	0.55
Minimum Differentiating Threshold	Awake	>1974 μV^2^	0.7275	0.85	0.60
Total Threshold-Crossing Frequency	All States	>860 μV^2^ crossed >62 times	0.9088	0.85	0.85
Total Threshold-Crossing Frequency	Awake	>86 μV^2^ crossed >1288 times	0.93	0.90	0.90
Hourly Threshold-Crossing Frequency	All States	>29 crossings/hour	0.8275	0.70	0.90
Hourly Threshold-Crossing Frequency	Awake	>1158 crossings/hour	0.82	0.55	1.00
Percent Time above Threshold	All States	>860 μV^2^ for >5.5% of EEG	0.8237	0.65	0.95
Percent Time above Threshold	Awake	>86 μV^2^ for >45% of EEG	0.7895	0.55	0.95

Abbreviations: AUC, Area Under the ROC Curve; IRDA, Intermittent Rhythmic Delta Activity. **Note:** Thresholds denote the minimum value needed to classify an individual as likely affected. “Frequency” corresponds to the total number of threshold-crossing events across all channels, while “Hourly Rate” normalizes these events per hour of EEG. “Percent Time above Threshold” reflects the fraction of the EEG that contains delta activity values above the specified threshold.

## Data Availability

Data is available from authors upon request due to privacy ethical restrictions.
